# Somatic cell count as an indicator of subclinical mastitis and increased inflammatory response in asymptomatic lactating women

**DOI:** 10.1128/spectrum.04051-23

**Published:** 2024-08-27

**Authors:** Angeliki Angelopoulou, Hugh M. B. Harris, Alicja K. Warda, Carol-Anne O'Shea, Aonghus Lavelle, C. Anthony Ryan, Eugene Dempsey, Catherine Stanton, Colin Hill, R. Paul Ross

**Affiliations:** 1 APC Microbiome Ireland, University College Cork, Cork, Ireland; 2 School of Microbiology, University College Cork, Cork, Ireland; 3 Department of Neonatology, Cork University Maternity Hospital, Cork, Ireland; 4 Department of Anatomy and Neuroscience, University College Cork, Cork, Ireland; 5 Food Biosciences, Teagasc Food Research Centre, Fermoy, Co Cork, Ireland; City of Hope Department of Pathology, Duarte, California, USA

**Keywords:** subclinical mastitis, clinical mastitis, somatic cells, somatic cell counts, breastfeeding, breast milk, lactation, milk microbiota, inflammation, interleukin-8

## Abstract

Subclinical mastitis is an asymptomatic inflammatory condition that can be difficult to define and diagnose. In the dairy industry, subclinical mastitis is diagnosed by milk somatic cell counts (SCCs) of ≥250,000 cells mL^−1^. In this pilot study, we assessed the efficacy of this index to identify human subclinical mastitis by comparing SCC levels with the inflammatory response [interleukin-8 (IL-8) levels] in 37 samples from asymptomatic and 10 clinical mastitis (CM) lactating women. The milk microbiota was determined by 16S rRNA gene sequencing. The SCC of CM samples ranged from 310,000 to 6,600,000 cells mL^−1^. However, 14 of 37 (37.8%) asymptomatic samples had high SCC (250,000–460,000 cells mL^−1^), indicating subclinical mastitis. SCC levels significantly (*P* < 0.001) and positively correlated with milk IL-8 levels reflecting the escalating inflammatory response across subclinical and clinical mastitis samples. Samples with an SCC of ≥250,000 cells mL^−1^ showed significant increases in IL-8 responses when compared with milk samples from healthy women. The milk microbiome of CM samples was dominated by streptococcal and staphylococcal species (89.9% combined median relative abundance). In contrast, the combined median streptococcal/staphylococcal relative levels were 75.4% and 66.3% in milks from asymptomatic (subclinical mastitis) and healthy groups, respectively. The *Streptococcus* genus was increased in samples with an SCC of ≥250,000, although this should be interpreted with caution. Thus, the index of ≥250,000 somatic cells mL^−1^ could be a reliable indicator of subclinical mastitis in humans and should aid future studies investigating the impact of subclinical mastitis on maternal health, breastfeeding behaviors, infant health, and development.

**IMPORTANCE:**

This pilot study suggests that SCC at a level of (greater than or equal to) 250,000 cells mL^−1^, as used in the dairy industry, is a suitable index to identify asymptomatic subclinical mastitis in lactating women since it reflects a significant increase in the inflammatory response compared to milk samples from healthy women. Using this index should aid studies into the short- and long-term consequences of subclinical mastitis for mother and infant.

## INTRODUCTION

Breastfeeding is the optimal regime for nearly all feeding newborn infants (1, 2) and simultaneously benefits the health of the mother-child dyad (3). Nonetheless, breastfeeding can be ceased due to breast inflammation, a condition known as mastitis (4), which has a frequency of up to 33% (5), with milk stasis and infection being the major causes (6). Lactational mastitis is classified into clinical and subclinical mastitis based on clinical manifestations (7). Clinical mastitis symptoms include breast redness, pain, pyrexia, flu-like symptoms, engorgement, and reduced milk secretion (7) with *Staphylococcus aureus* being the major pathogen identified. In contrast, subclinical mastitis occurs more frequently than clinical mastitis and is an often misidentified entity (8). It is prevalent during early lactation, often with absence of symptoms (9), and is usually associated with coagulase-negative staphylococci (5). However, when symptoms are reported, these include sharp, needling pain and a burning sensation (7). Importantly, subclinical mastitis in humans is associated with dramatic changes in the inflammatory/anti-inflammatory profiles of breast milk (8). Diagnosis of subclinical mastitis in the absence of symptoms can be achieved by measuring sodium/potassium (Na^+^:K^+^) ratio and interleukin-8 (IL-8) levels in breast milk (9). The proinflammatory cytokine IL-8 is produced within the mammary gland and can be easily measured in milk (10, 11). Along with other cytokines, IL-8 mediates tight junction-permeabilization between the epithelial cells, thus allowing plasma constituents, including Na^+^, to cross into milk (12). Thus, a Na^+^:K^+^ ratio of >0.6 and increased IL-8 levels are considered indicators of subclinical mastitis in humans (9, 13). However, subclinical mastitis in humans remains challenging to identify and therefore define. Despite this, subclinical mastitis has been identified as a risk factor for HIV transmission between mother and infant (9) and poor weight gain/growth in infants (10, 11, 14–17). In a European multicenter cohort, subclinical mastitis has also been shown to alter the composition of breast milk by significantly reducing the concentrations of lactose, docosahexaenoic acid (DHA), linolenic acid, calcium, and phosphorus while significantly increasing total protein, α-lactalbumin, albumin, arachidonic acid to DHA ratio, *n-6* to *n-3* fatty acid ratio and several minerals (18). The consequences of these alterations on breast milk output of the mother, breastfeeding behaviors, and infant growth and development are unknown. A reliable indicator of subclinical mastitis could help in the progression of such research.

In a study investigating the associations between human clinical mastitis and common indices of mammary gland inflammation, namely, IL-8, Na^+^ levels, and somatic cell counts (SCCs), Hunt et al. (19) concluded that IL-8 and SCC may be better indicators of mammary inflammation. Indeed, while IL-8 and SCC levels were both significantly elevated in milk from symptomatic breasts (IL-8 = 2,960 pg mL^−1^; SCC = 1,564,000 cells mL^−1^) compared with those from non-symptomatic breasts (IL-8 = 302 pg mL^−1^; SCC = 120,000 cells mL^−1^), Na^+^ only “tended” to be greater in milk from symptomatic breasts (7.3 mM versus 5.0 mM in asymptomatic samples). SCC is the standard method used in the dairy industry to diagnose mastitis and to assess the immunological status of lactating cows (20), where subclinical mastitis in cows is associated with changes in milk quality (21). As in humans, IL-8 is also higher in milk from cows with subclinical mastitis (21). In another study, the positive linear relationship observed between SCC and total bacterial counts led the authors to conclude that SCC should be used as a diagnostic tool for preliminary assessment of lactating breast health (22). However, Wren et al. (23) concluded that SCC may not be a suitable biomarker of subclinical mastitis in lactating women, given that it did not correlate with the Na^+^:K^+^ ratio or proinflammatory cytokines at two stages of lactation.

Given the paucity of data on SCC levels in human subclinical mastitis, we set out to assess the suitability of using the dairy index for subclinical mastitis, ≥250,000 cells mL^−1^ (24, 25), to identify subclinical mastitis in asymptomatic women. We, therefore, analyzed the SCC levels in breast milk samples from asymptomatic lactating women along with samples from women diagnosed with clinical mastitis (CM). After stratifying the samples as healthy (H), subclinical mastitis (SM), and CM, we evaluated the IL-8 levels across all milk samples to assess the extent of the inflammatory response and the suitability of using SCC to identify samples with potential subclinical mastitis. Finally, we examined the microbiota profiles of all milk samples to determine microbiological differences/similarities between them.

## MATERIALS AND METHODS

### Design

This is a cross-sectional descriptive study that was designed to assess the microbiome and immunological profiles of milk from healthy, subclinical, and clinical mastitic lactating mothers.

### Setting

The pilot study was carried out from June 2017 to May 2019. The participating women were recruited by Cork University Maternity Hospital, Cork, Ireland.

### Data collection

Demographic data were not collected since the aim of the pilot study was to determine the microbiome and immunological profiles of milk from healthy, subclinical, and clinical mastitic lactating mothers. The inclusion criteria were that the samples should be collected during the fourth week post-partum and the mothers who were diagnosed with mastitis (CM) should display local (breast redness, engorgement of breast, and pain) and/or systemic symptoms (pyrexia and flu-like symptoms). The exclusion criterion was the administration of antibiotics before sample collection in the previous 4 weeks. Forty-seven lactating mothers were recruited, from which 10 were diagnosed with mastitis.

### Sample collection

A detailed explanation of the sampling procedure was given to the participating women by the research nurses and samples were self-collected. Nipples and mammary areola were cleaned with sterile alcohol-free aqueous solution wipes (Ted Kelleher, First Aid & Hygiene Supplies Ltd, Macroom, Ireland). Hand expression was recommended, but pumping was also acceptable. Approximately, 10 mL of milk was aseptically collected using sterile gloves in a sterile tube with the first few drops (~500 µL) being discarded. Within 24 hours after the receipt of samples, 125 µL of each milk sample was used for SCC enumeration. Samples were stored below 4°C until processing and remaining volumes were immediately frozen at –20°C for subsequent DNA extraction and measurement of IL-8.

### Measurement

#### SCC enumeration

We employed a direct microscopic method (DMSCC) using the single-strip counting procedure (26) allowing for enumeration of SCC in the limited volume of the acquired samples. In brief, three smears (10 µL each) were prepared on somatic cell slides (5638–01930; Bellco Glass Inc., Vineland, NJ, USA) and air-dried overnight. Smears were stained by adding two drops of modified Newman-Lampert Stain solution (Sigma-Aldrich, Germany). Following 2-min incubation, excess stain was removed by resting the edge of the slide on absorbent paper and air-drying. The slides were dipped thrice in water at 37°C–45°C. Somatic cells were enumerated on each of the smears using a light microscope at ×100 magnification. The field diameter (fd) was measured with a stage micrometer to calculate the single-strip factor (SSF) according to the formula SSF = 10,000/(11.28 × fd). The SSF was multiplied by each strip count to determine SCC mL^−1^.

To validate this method, we analyzed the SCC of 12 fresh bovine milk samples using a Somacount 300 (Bentley Instruments, Inc., Chaska, MN, USA) and the DMSCC method, as described above.

#### Criteria for sample classification

Samples were classified as H if their SCC was ˂ 250,000 cells mL^−1^. Milk samples were classified as subclinical (SM) when they were originating from an asymptomatic donor and their SCC was ≥250,000 cells mL^−1^ following the classification criteria used in the dairy industry (24, 25). For comparative purposes, a subset of 10 H and 10 SM samples (matching the number of CM samples) was selected using a random number generator.

#### IL-8

Two milliliters of the milk samples was centrifuged once at 10,000 × *g* for 30 min at 4°C. The fat layer was bypassed using a thin needle (Sterican, B. Braun, Ireland) and the liquid phase underneath was extracted for further processing. The IL-8 human ELISA kit (Invitrogen, ThermoFisher Scientific, Waltham, MA, USA) was used according to the manufacturer’s instructions. Absorbance was read at 450 nm using a microtiter plate reader (Spectramax M3; Molecular Devices, San Jose, CA, USA). Based on the standard curve, the IL-8 levels in each test sample were quantitated. IL-8 was quantified for 7 out of 10 CM samples due to limited volume of these samples.

#### DNA extraction and 16S rRNA gene sequencing

DNA was purified from milk samples using the PowerFood Microbial DNA Isolation Kit (MoBio Laboratories, Carlsbad, USA) as previously described (27). PCR amplification of the V3–V4 region was performed using the forward primer 5′-TCGTCGGCAGCGTCAGATGTGTATAAGAGACAG*CCTACGGG**NGGCWGCAG*-3′ and the reverse primer 5′-GTCTCGTGGGCTCGGAGATGTGTATAAGAGACAG*GACTAC**HVGGGTATCTAATCC*-3′. Each 30-µL PCR reaction contained up to 5 ng µL^−1^ microbial genomic DNA, 1 µL of each primer (6 µM), and 15-µL Phusion High-Fidelity PCR Master Mix (ThermoFisher Scientific). The PCR conditions used were identical to the ones used by Angelopoulou et al. (27). The Agencourt AMPure XP system (Beckman Coulter, UK) was used to purify the amplicons. A subsequent limited‐cycle amplification step was performed to add multiplexing indices and Illumina sequencing adapters. Amplicons were quantified, normalized, and pooled using the Qubit dsDNA HS Assay Kit (Life Technologies, Carlsbad, CA, USA). Library preparation was carried out by GATC Biotech (Ebersberg, Germany) prior to 2 × 300 bp sequencing on the Illumina MiSeq platform.

### Bioinformatic analysis of high-throughput sequencing data

Raw reads were quality checked using fastqc (v.0.11.5, http://www.bioinformatics.babraham.ac.uk/projects/fastqc). Primers were removed using cutadapt (v) (28). Amplicon sequence variants (ASVs) were inferred using the dada2 pipeline (29) implemented in R (https://www.r-project.org/). Taxonomy assignment was performed using the SILVA reference database (v.138.1) (30). ASVs not assigned to a phylum were removed.

### Data analysis

All statistics for bioinformatic analysis were performed in R (v.3.6.1). The phyloseq package (v.1.40.0) (31) was used for storing and managing microbiome data. Alpha diversity was calculated using the number of observed species and the Shannon entropy. Beta diversity was calculated on total sample sum normalized data using the Bray-Curtis divergence. Permutational multivariate analysis of variance was used to test grouping variables using 999 simulations. The vegan package (v.2.6–2) was used for calculating diversity metrics. Differential abundance was calculated using linear discriminant analysis with effect size (LEfSe) using default settings (32). Pairwise differences between levels within grouping variables (asymptomatic, subclinical mastitis and clinical mastitis; SCC of <250,000 and >250,000 were compared using the Wilcoxon signed-rank test. Plotting was performed using the ggplot2 (33) and ggpubr (34) packages. IL-8 and SCC data were not normally distributed, as assessed by the Shapiro-Wilk test, and were compared by pairwise Wilcoxon tests. Correlation of IL-8 and SCC data was performed with the Spearman method. Correlations between DMSCC and automated methods were performed with the Pearson method. For all tests, differences were considered significant at a *P* value of ≤0.05.

## RESULTS

### Based on SCC, 37.8% of asymptomatic samples were subclinical

**Fig 1:**
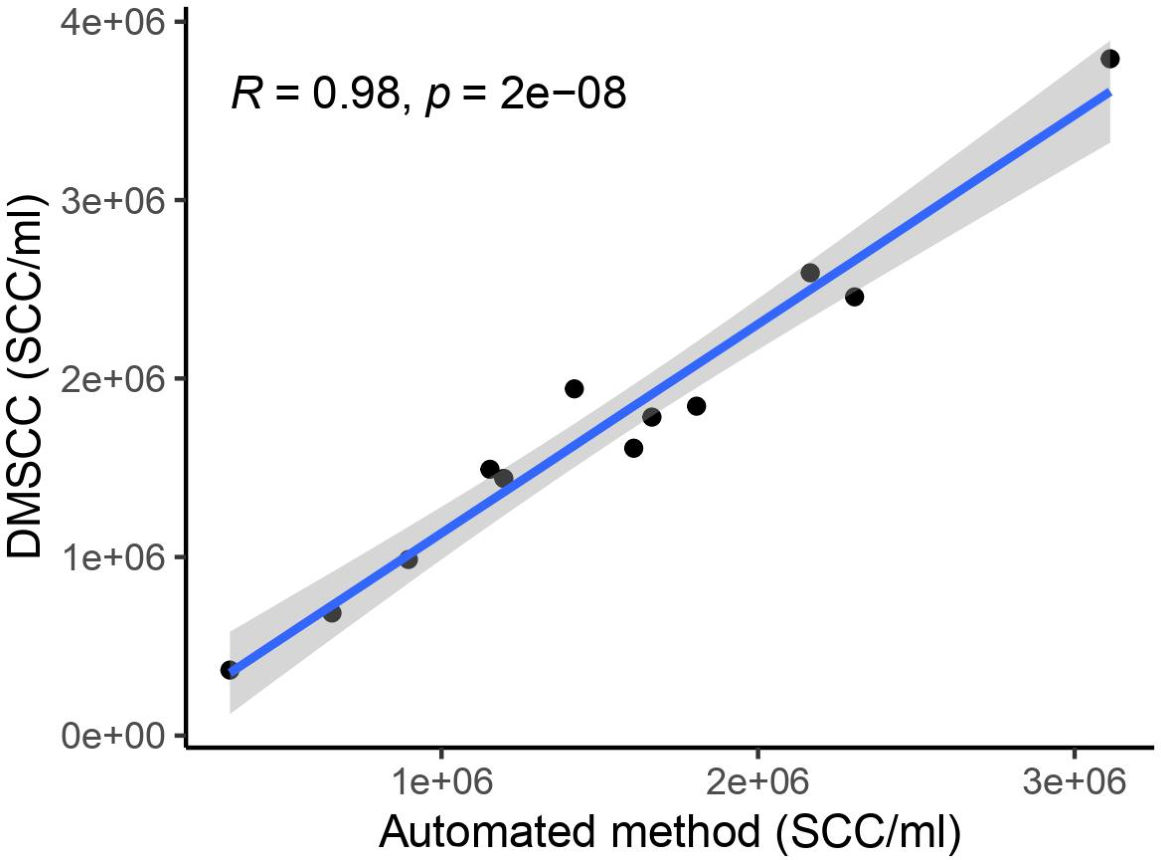
Pearson correlation of automated method versus DMSCC for the enumeration of somatic cells in 12 fresh bovine milk samples (*R* = 0.98). DMSCC, direct microscopy somatic cell count; SCC, somatic cell count.

For SCC enumeration, we initially compared the automated method and the DMSCC method using fresh bovine milk samples. As both methods provided similar counts with the two methods strongly correlating with each other (*R* = 0.98, Fig. 1), we proceeded with DMSCC to analyze our human milk samples.

**Fig 2:**
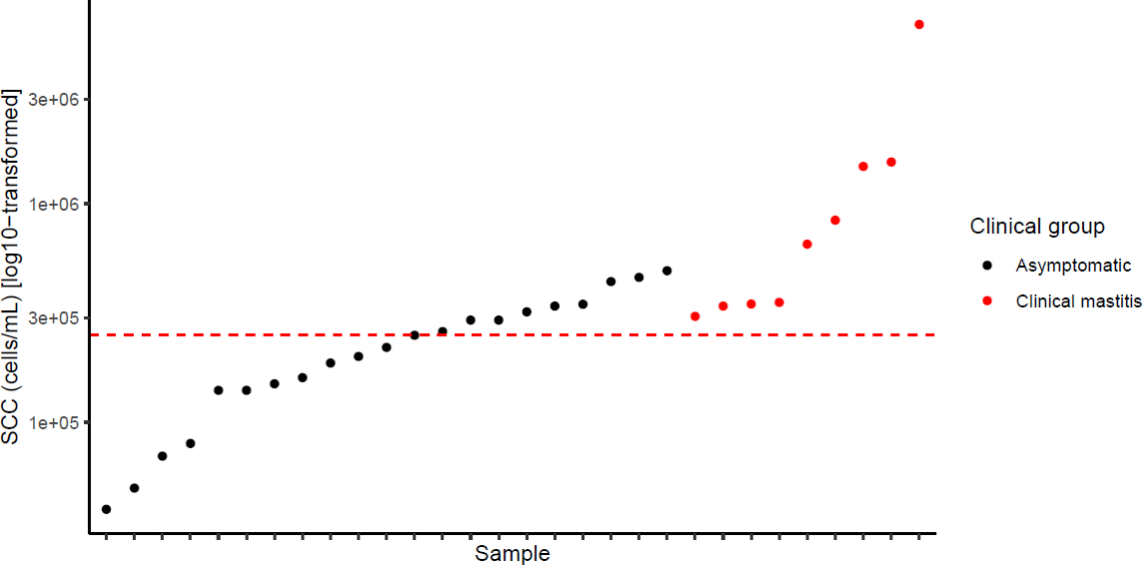
SCC of 37 asymptomatic (●) milk samples and 10 clinical mastitic (●) milk samples. The red line indicates the cut-off for SCC of ≥250,000 cells mL^−1^.

**Fig 3:**
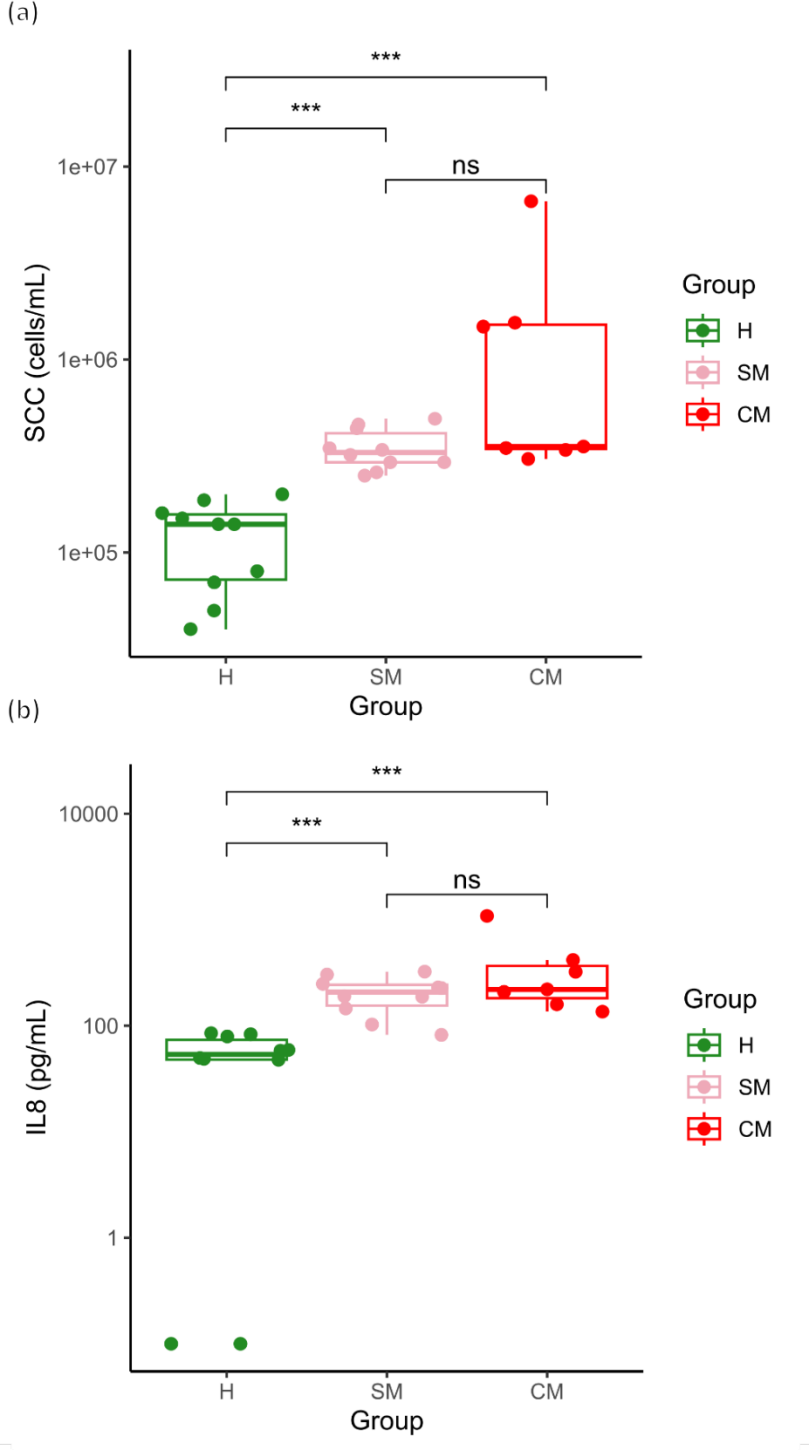
Boxplots of SCC (a, cells mL^−1^) enumeration and IL-8 (b, pg mL^−1^) concentration in healthy (H, green), subclinical (SM, pink), and clinical (CM, red) mastitis milk samples (pairwise Wilcoxon test; ***P < 0.001). SCC, somatic cell count.

In total, 47 breast milk samples were analyzed, 37 of which were collected from asymptomatic women and 10 of which were from women diagnosed with clinical mastitis. The SCC of the asymptomatic samples (A samples, Fig. 2) ranged from 35,000 to 490,000 cells mL^−1^, whereas the SCCs of the clinical mastitis samples were between 310,000 and 6,600,000 cells mL^−1^ (CM samples, Fig. 2). Applying the criteria for subclinical mastitis (SCC ≥250,000 cells mL^−1^) to the asymptomatic samples revealed that 37.8% (*n* = 14) of the asymptomatic samples were potentially subclinical (Table 1). Hereafter, the samples were referred to as H, SM, and CM. Median SCC levels (cells mL^−1^) with interquartile range (IQR) are presented for each group in Fig. 3a. A significant difference (*P* < 0.001) was observed between the SCC for H samples (median 140,000 IQR 85,000) and SM samples (median 330,000, IQR 123,334). Moreover, a significant difference (*P* < 0.001) was detected between H and the CM group (median 353,614; IQR 1,171,306). No significant difference was observed between SM and CM samples.

**TABLE 1:** SCC-based classification of milk samples from asymptomatic women (A), women with subclinical mastitis (SM), and those with clinical mastitis (CM)

State	SCC	Sample classification based on SCC
A	190,000	H1
A	140,000	H2
A	70,000	H3
A	40,000	H4
A	140,000	H5
A	80,000	H6
A	50,000	H7
A	200,000	H8
A	160,000	H9
A	150,000	H10
A	220,000	H11
A	40,000	H12
A	120,000	H13
A	35,000	H14
A	50,000	H15
A	50,000	H16
A	160,000	H17
A	49,000	H18
A	40,000	H19
A	190,000	H20
A	160,000	H21
A	80,000	H22
A	230,000	H23
A	260,000	SM1
A	440,000	SM2
A	340,000	SM3
A	250,000	SM4
A	490,000	SM5
A	250,000	SM6
A	350,000	SM7
A	460,000	SM8
A	320,000	SM9
A	290,000	SM10
A	420,000	SM11
A	360,000	SM12
A	310,000	SM13
A	430,000	SM14
CM1	1,600,000	CM1
CM2	340,000	CM2
CM3	840,000	CM3
CM4	6,600,000	CM4
CM5	1,500,000	CM5
CM6	650,000	CM6
CM7	310,000	CM7
CM8	350,000	CM8
CM9	800,000	CM9
CM10	920,000	CM10

### IL-8 levels in breast milk samples correlate with SCC results

**Fig 4:**
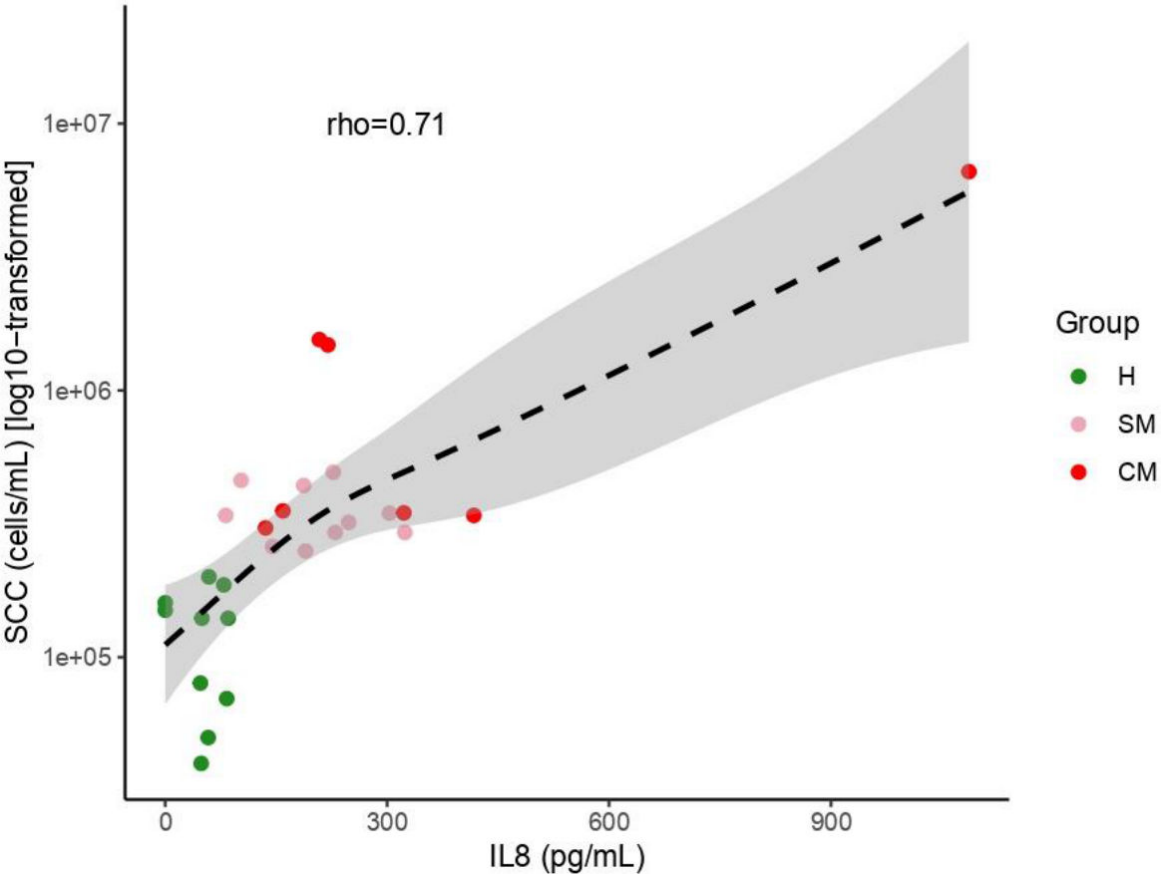
Relationship between SCC (cells/mL) and IL-8 (pg/mL) in milk samples with H (green, *n* = 10) being healthy samples, SM (pink, *n* = 10) subclinical mastitis samples and CM, clinical mastitic samples (red, *n* = 7). Regression line was plotted using the “gam” function, and the Spearman correlation coefficient is reported.

Given that IL-8 levels in breast milk are considered a reliable indicator of subclinical mastitis, we next measured IL-8 levels in all milk samples and determined if the levels observed correlated with our classification of milk samples based on SCC enumeration. We detected a significant positive correlation between the two (rho = 0.71; *P* < 0.001; Fig. 4). Moreover, after removing the highest outlier, we still observed a significant correlation (rho = 0.68; *P* < 0.001) between SCC and IL-8.

Median IL-8 levels (pg mL^−1^) with IQR are presented in Fig. 3b. The concentrations of IL-8 for two of the tested H samples were below the detection limit and thus are presented as 0.1 pg mL^−1^ (Fig. 3b). A significant difference (*P* < 0.001) was observed between H (median 53.9, IQR 26.4) and SM samples (median 208.5, IQR 87.7). Furthermore, we detected a significant difference (*P* < 0.001) between H and CM samples (median 219.99, IQR 186.2) (Fig. 3b). No significant difference was observed between SM and CM samples.

### 16S rRNA gene sequencing identified a common milk microbiota in more than 50% of samples

**Fig 5:**
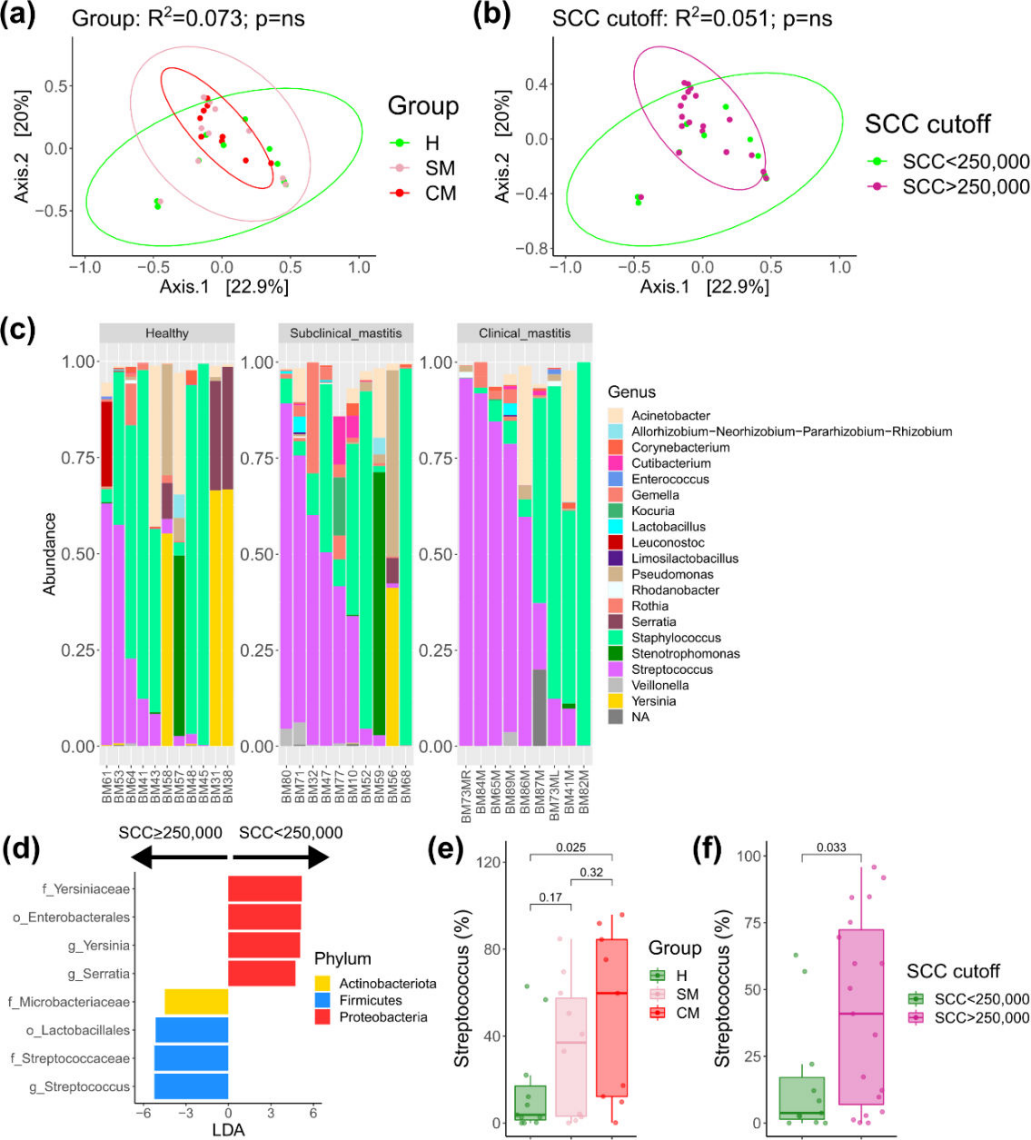
Beta diversity PCoA plots of Bray-Curtis divergence for (**a**) groups H, SM, and CM and (**b**) SCC cut-off of 250,000 cells. (**c**) Taxonomic barplots at the genus level of the top 20 most abundant genera. (**d**) LEfSe results comparing SCC cut-off of 250,000 cells. Boxplots of *Streptococcus* genus abundance between groups of H, SM, and CM (**e**) and SCC cut-off (**f**). CM, clinical mastitis; H, health; SCC, somatic cell count; SM-Subclinical mastitis.

Amplicon sequencing of human milk samples was available for 30 samples [H (*n* = 11), SM (*n* = 10), CM (*n* = 9)]. One CM sample failed sequencing. Seven samples were re-sequenced on a second sequencing run due to low read counts on the first run. Sequencing yielded a total of 1,062,261 reads post-processing with median reads per sample of 16,869.5 reads (IQR 5390). Breast milk samples were dominated by *Streptococcus*, *Acinetobacter*, *Staphylococcus*, *Pseudomonas*, *Rothia*, *Corynebacterium*, *Cutibacterium*, and *Gemella*, with these genera in greater than 50% of samples (Fig. S1). We did not observe significant differences for the alpha (Fig. S2a and b) and beta diversity metrics (Fig. 5a and b) for comparisons between the H, SM, and CM groups or the groups based on a cut-off of 250,000 SCCs. There was also no significant difference between sequencing runs (Fig. S2c).

However, a trend for increased *Streptococcus* in subjects with clinical and subclinical mastitis was suggested (Fig. 5c), with an increase in the combined percentage of *Streptococcus* and *Staphylococcus* from H [66.3% (IQR 90%)], SM [75.4% (IQR 38.3%)], and CM [89.9% (IQR 22.9%)]. However, as can be seen by the interquartile ranges, there was very large variability between samples, particularly in the H group, so we performed differential abundance testing, comparing subjects with an SCC of ≥250,000 (equivalent to combining SM and CM) versus those with an SCC of <250,000 (equivalent to the H group). This demonstrated an increase in the genus *Streptococcus* in subjects with an SCC of ≥250,000 (Fig. 5d). Interestingly, *Yersinia* and *Serratia* were increased in subjects with an SCC of <250,000, although this was driven by three samples in the H group. When looking specifically at *Streptococcus* percentage, there was an increasing trend from H to SM, to CM (Fig. 5e) which accounted for the difference based on SCC cut-off (Fig. 5f). Correlating *Streptococcus* counts with SCC demonstrated a positive correlation which did not pass the significance threshold (Spearman rho 0.359, *P* = 0.051). There was no correlation between *Streptococcus* and IL-8 (Spearman rho 0.18, *P* = 0.33).

## DISCUSSION

International health organizations encourage women to breastfeed during the first 6 months of life (35). However, mastitis is a major cause of breastfeeding cessation. Subclinical mastitis is generally an asymptomatic inflammatory condition that is more common than clinical mastitis yet difficult to define. Despite this, subclinical mastitis can negatively impact infant health (9–11, 14–18). Thus, in this pilot study, we used SCC to stratify human milk samples into H, SM, and CM based on the dairy index for subclinical mastitis, ≥250,000 cells mL^−1^ (24, 25). Of the 47 milk samples, 10 were from mothers with diagnosed clinical mastitis.

Subclinical mastitis was observed in 37.8% of asymptomatic women based on SCC enumeration, which is in agreement with the results of Samuel et al. (18), who measured the Na^+^:K^+^ ratio in the milk of 305 women with 35.4% of the women having at least one episode of subclinical mastitis. The SCC for SM and CM samples were significantly different from the H samples, though no significant difference was observed between SM and CM samples. To determine if the increased somatic cells were indicative of increased inflammation, we measured levels of IL-8, a cytokine produced to elicit the infiltration of immune cells and commonly used as an inflammation index of mammary gland inflammation (8, 19) in both bovine and human milk (10, 19, 36). In our pilot study, we detected significant differences in IL-8 levels between H and SM samples (*P* < 0.001) and H and CM samples (*P* < 0.001) (though not between SM and CM samples), indicating the presence of inflammation in SM and CM samples. Indeed, SCC correlated significantly and positively with IL-8 levels in our samples with the increase of SCC in SM and CM samples implying a significant immune response. This is in agreement with the findings of Hunt et al. (19), who detected a 10-fold increase of IL-8 and SCC in mastitic milk samples compared to healthy milk samples. Thus, an SCC of ≥250,000 cells mL^−1^ in human breast milk could potentially be used as a diagnostic tool for subclinical mastitis. Given the unreliability of using Na^+^ levels in milk for identifying mammary inflammation (19), the significant correlation between SCC and IL-8 levels identified in this study and that of Hunt et al. (19), as well as the universal use of the dairy index for identifying subclinical mastitis in the dairy industry (24, 25), SCC may prove more advantageous than previous indicators of human mastitis.

We next assessed the microbiota profiles of the H, SM, and CM milk samples. Amplicon 16S rRNA sequencing revealed that eight genera (*Streptococcus*, *Acinetobacter*, *Staphylococcus*, *Pseudomonas*, *Rothia*, *Corynebacterium*, *Cutibacterium*, and *Gemella*) were present in over 50% of the samples. Three of these have been identified as members of the “core” milk microbiota (*Streptococcus*, *Staphylococcus*, and *Pseudomonas*) by Murphy et al. (37), who reported 12 core genera present in 90% or more of the study subjects (*n* = 10). Likewise, Hunt et al. (38) reported nine core OTUs (present in 100% of subjects, *n* = 9) constituting the core microbiota that also included *Streptococcus*, *Staphylococcus*, *Pseudomonas*, as well as *Corynebacterium*. The observed differences between the three studies could reflect the diversity of milk samples due to geographical location (39, 40) or other factors.

No statistically significant differences were observed in alpha diversity or beta diversity based on clinical groups or cut-off points. Using LEfSe, we did observe an increase in the genus *Streptococcus* in samples with an SCC of ≥250,000, with increasing abundance from H to SM, to CM. *Streptococcus* is a core member of the human milk microbiota, and *Streptococcus* spp. are dominant commensals in the oral cavity of infants (41). Due to the relatively small numbers in each group and the weak and non-significant correlation of *Streptococcus* with SCC counts, this finding must be interpreted with caution. Moreover, challenges related to analyzing low biomass samples such as human milk (38, 42) and the polymicrobial nature of mastitis need to be considered. However, Bolte et al. (43) reported that *Streptococcus* spp. were associated with a diet high in animal protein and fat and were increased in subjects with inflammatory bowel disease and colon cancer, among other diseases. Future studies with larger sample sizes are needed to verify our findings to determine if increased *Streptococcus* abundance is a feature of the subclinical milk microbiome and if this is contributing to the proinflammatory state.

Interestingly, samples with an SCC of ≥250,000 had lower levels of *Serratia*/*Yersinia* compared to H samples. *Serratia* was a member of the core genera in healthy human milk samples in the study conducted by Hunt et al. (38). In contrast, other studies have identified *Serratia* in bovine (44) and human mastitis (45). In line with our observations, *Pseudomonas* has been reported as a dominant member of the human milk microbiome in several studies (38, 46, 47), and the same applies to *Staphylococcus* (38, 45, 48–51) and *Streptococcus* (5, 45, 52). Interestingly, *Staphylococcus* and *Streptococcus* species constituted 82% of species in CM milk samples, but their combined relative levels were less in SM and H samples, at 60% and 45%, respectively.

### Limitations

We must acknowledge some limitations in this pilot study, the first being the heterogeneity of the symptoms of the cases when the samples were collected. In addition, data are also limited in that only basic information regarding the symptomatology was collected. Therefore, information about a mother’s background and demographics was not available to single out other factors that might affect breastfeeding practices and SCC levels during mastitis such as parity, lactation status, age, sampling time, feeding frequency, and season. However, this pilot study should form the basis for follow-up studies with larger sample sizes.

### Conclusions

Using the dairy index for subclinical mastitis (≥250,000 cells mL^−1^), we stratified human milk samples from women who were asymptomatic for mastitis or diagnosed with clinical mastitis into H, SM, and CM. IL-8 analysis of the milk samples verified our approach as the increasing SCC in SM and CM samples correlated with an escalating immune response. A high prevalence (37.8%) of subclinical mastitis was found in the population of 37 asymptomatic lactating women. More research into the consequences of subclinical mastitis for infant and maternal health is clearly warranted. The significance of this pilot study is that SCC at a level of ≥250,000 cells mL^−1^ is a suitable index to identify asymptomatic subclinical mastitis in lactating women since it reflects a significant increase in the inflammatory response compared to milk samples from healthy women and should aid studies into the short- and long-term consequences of subclinical mastitis for mother and infant.

## Data Availability

Sequencing data are available at the Sequence Read Archive under the BioProject ID PRJNA1141436. All analysis codes, a phyloseq object with processed sequencing data, and all other data presented in the paper are accessible at https://github.com/ajlavelle/human_mastitis_study.

## SUPPLEMENTAL MATERIAL

Figure S1 spectrum.04051-23-s0001.tiff Prevalence and mean abundance of genera in breast milk.

Figure S2 spectrum.04051-23-s0002.tiff Observed ASVs and Shannon entropy.

ASM does not own the copyrights to Supplemental Material that may be linked to, or accessed through, an article. The authors have granted ASM a non-exclusive, world-wide license to publish the Supplemental Material files. Please contact the corresponding author directly for reuse.
