# Supplementary figures and images for: Somatic cell count as an indicator of subclinical mastitis and increased inflammatory response in asymptomatic lactating women
Source: Microbiol Spectr. Author manuscript; Available in PMC 2024 Oct 4. (PMC11448179; doi:10.1128/spectrum.04051-23)

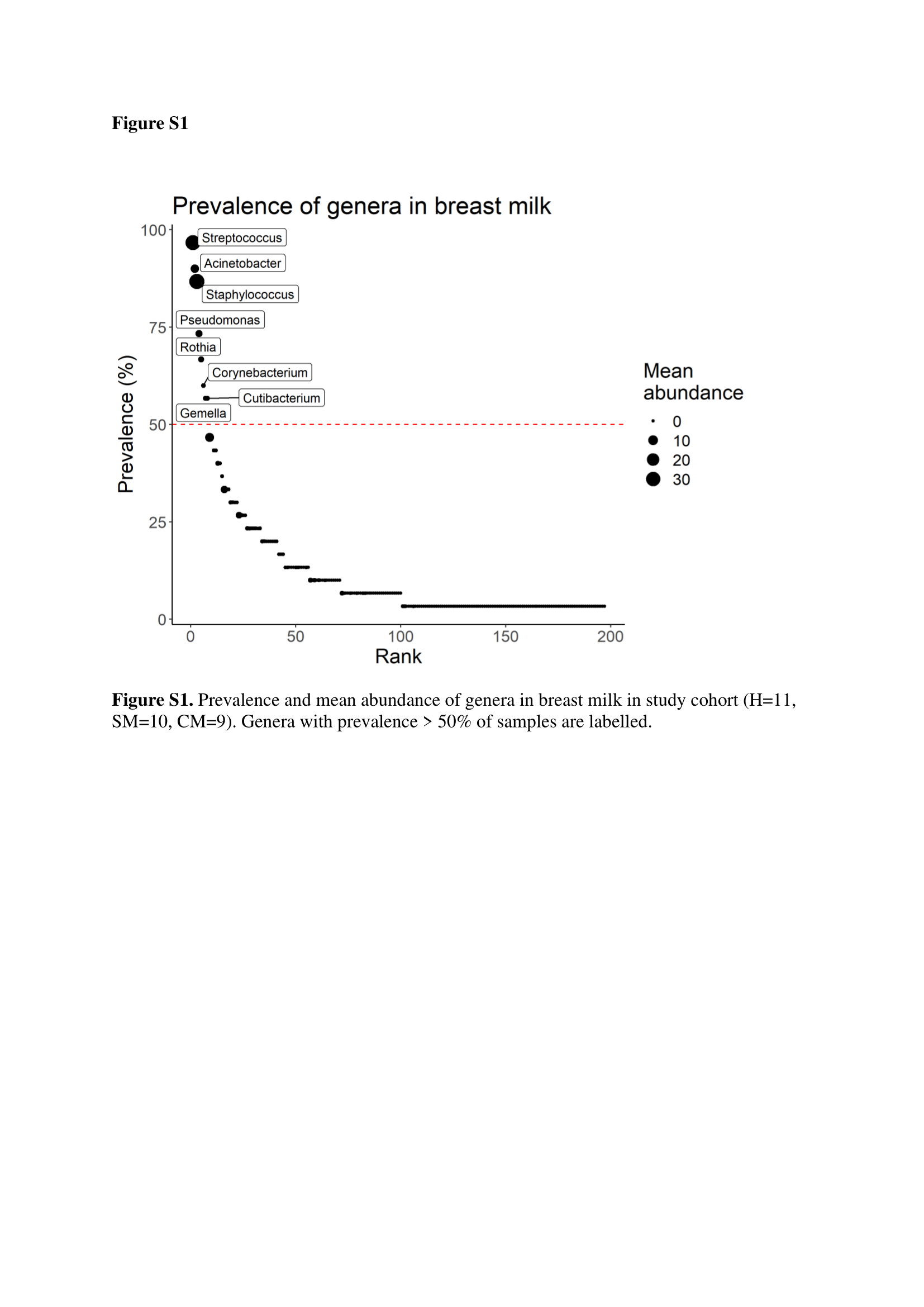

Supplement: Figure S1 - Prevalence and mean abundance of genera in breast milk. [file EMS198463-supplement-Figure_S1___Prevalence_and_mean_abundance_of_genera_in_breast_milk_.tiff]

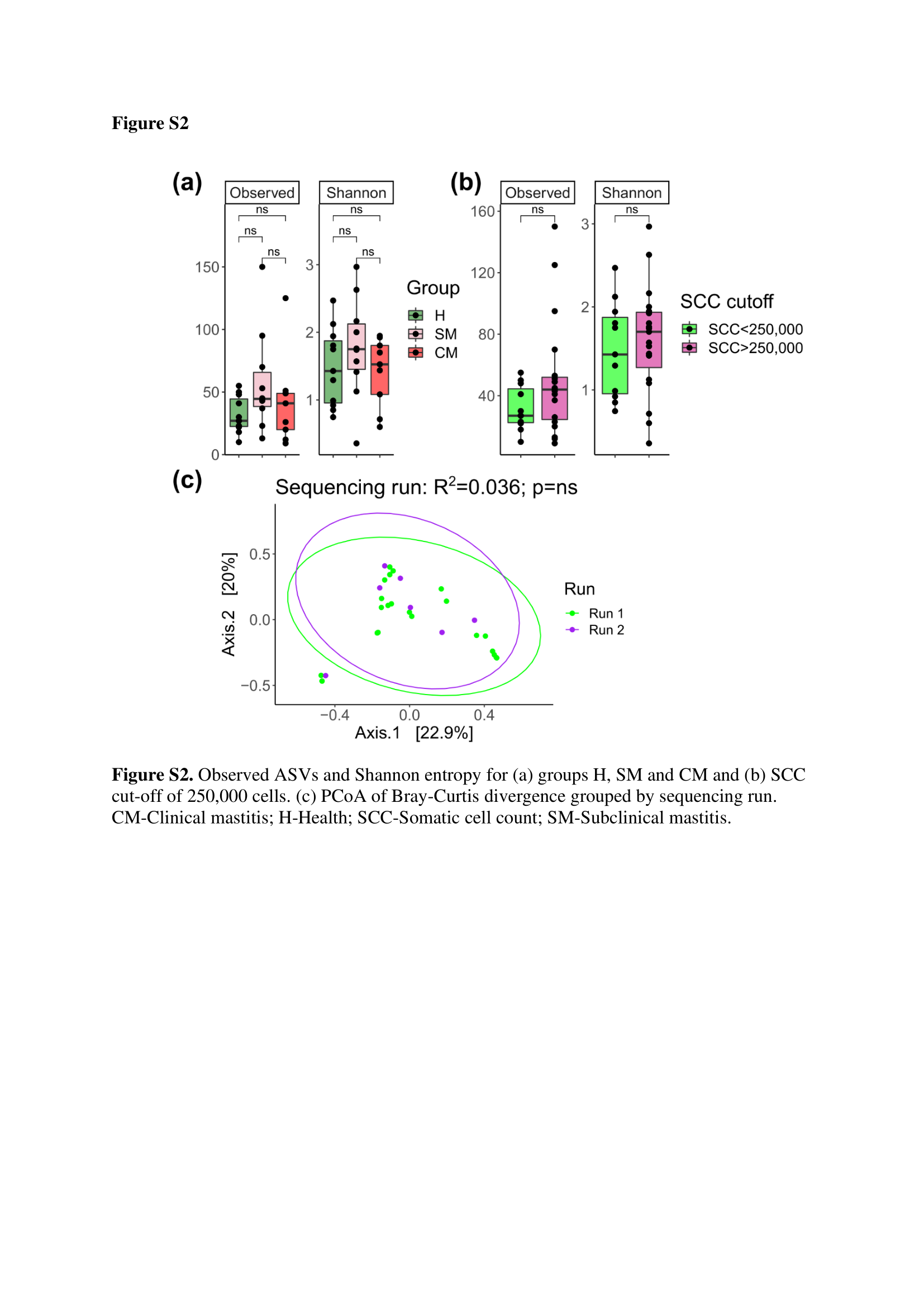

Supplement: Figure S2 - Observed ASVs and Shannon entropy. [file EMS198463-supplement-Figure_S2___Observed_ASVs_and_Shannon_entropy_.tiff]
